# Rational Design, Stabilities and Nonlinear Optical Properties of Non-Conventional Transition Metalides; New Entry into Nonlinear Optical Materials

**DOI:** 10.3390/ma16093447

**Published:** 2023-04-28

**Authors:** Mohammed A. Alkhalifah, Nadeem S. Sheikh, Yasair S. S. Al-Faiyz, Imene Bayach, Ralf Ludwig, Khurshid Ayub

**Affiliations:** 1 Department of Chemistry, College of Science, King Faisal University, Al-Ahsa 31982, Saudi Arabia; 2 Chemical Sciences, Faculty of Science, Universiti Brunei Darussalam, Jalan Tungku Link, Gadong BE1410, Brunei; 3 University of Rostock, Institute of Chemistry, Physical and Theoretical Chemistry, Albert-Einstein-Straße 27, 18059 Rostock, Germany; 4 Department of Science and Technology of Life, Light and Matter, Faculty of Interdisciplinary Research, University of Rostock, 18059 Rostock, Germany; 5 Leibniz-Institut für Katalyse an der Universität Rostock, Albert-Einstein-Strasse 29a, 18059 Rostock, Germany; 6 Department of Chemistry, COMSATS University Islamabad, Abbottabad Campus, Abbottabad 22060, KPK, Pakistan

**Keywords:** transition metalide, X_12_Y_12_ nanoclusters, hyperpolarizability, electrides, density functional theory

## Abstract

Electronic and nonlinear optical properties of endohedral metallofullerenes are presented. The endohedral metallofullerenes contain transition metal encapsulated in inorganic fullerenes X_12_Y_12_ (X = B, Al & Y = N, P). The endohedral metallofullerenes (**endo-TM@X_12_Y_12_**) possess quite interesting geometric and electronic properties, which are the function of the nature of the atom and the size of fullerene. NBO charge and frontier molecular orbital analyses reveal that the transition metal encapsulated Al_12_N_12_ fullerenes (**endo-TM@Al_12_N_12_**) are true metalides when the transition metals are Ni, Cu and Zn. **Endo-Cr@Al_12_N_12_** and **endo-Co@Al_12_N_12_** are at the borderline between metalides and electrides with predominantly electride characteristics. The other members of the series are excess electron systems, which offer interesting electronic and nonlinear optical properties. The diversity of nature possessed by **endo-TM@Al_12_N_12_** is not prevalent for other fullerenes. **Endo-TM@Al_12_P_12_** are true metalides when the transition metals are (Cr-Zn). HOMO-LUMO gaps (E_H-L_) are reduced significantly for these endohedral metallofullerenes, with a maximum percent decrease in E_H-L_ of up to 70%. Many complexes show odd–even oscillating behavior for E_H-L_ and dipole moments. Odd electron species contain large dipole moments and small E_H-L,_ whereas even electron systems have the opposite behavior. Despite the decrease in E_H-L_, these systems show high kinetic and thermodynamic stabilities. The encapsulation of transition metals is a highly exergonic process. These **endo-TM@X_12_Y_12_** possess remarkable nonlinear optical response in which the first hyperpolarizability reaches up to 2.79 × 10^5^ au for **endo-V@Al_12_N_12_.** This study helps in the comparative analysis of the potential nonlinear optical responses of electrides, metalides and other excess electron systems. In general, the potential nonlinear optical response of electrides is higher than metalides but lower than those of simple excess electron compounds. The higher non-linear optical response and interesting electronic characteristics of **endo-TM@Al_12_N_12_** complexes may be promising contenders for potential NLO applications.

## 1. Introduction

Alkalides are the compounds where anionic positions are occupied by alkali metal anions [1]. Alkalides are the focus of research in the current decade due to their fascinating properties including the theoretically predicted large nonlinear optical response [2,3,4,5,6,7,8,9,10]. The concept of alkalides has been well known for more than five decades now both in solution [11,12,13,14] and crystalline phase [15,16]; however, the room-temperature stable alkalides were reported by Dye and co-workers in 1999 [17]. A number of reports are available in the literature which illustrate the large nonlinear optical response of alkalides. For example, Wei Chen et al. [18] reported that the hyperpolarizability of AdzLi^+^Na^−^ is remarkably high (*β_o_* = 6.20681 × 10^5^ au). Similarly, an increase in the nonlinear optical response of Li^+^(calix[4]pyrrole)M^−^ (M = Li, Na and K) is also reported by the introduction of alkalides [7]. The hyperpolarizability values of these alkalides are larger than their corresponding electrides [7]. Similarly, the alkalides from organic-cage-like compounds are also reported where alkali metal cation resides inside the cage, whereas the alkalide resides outside the cage [7]. The nonlinear optical response of these alkalides [9] is even higher than the nonlinear optical response of inverse metal hydride and Li^+^(calix[4]pyrrole)M^−^ (M = Li, Na and K). More recently, Janus alkalides are reported from facially polarized Janus all-*cis*-1,2,3,4,5,6-hexafluorocyclohexane. The hyperpolarizability values of these alkalides [10] (calculated at MP2/6-31+G(d,p) reach up to 1.45 × 10^6^ au.

All of the alkalides reported in the literature share a common design principle. A complexant interacts with two atoms (either both alkali metal atoms or an alkali and electropositive atom) where one atom receives a positive charge (alkali or other electropositive atom), while the second alkali metal atom receives a negative charge. For example, in the reverse metal hydride [18,19], a hydrogen atom receives a positive charge upon interaction with nitrogen atoms, whereas the alkali metal atom receives negative charge. For a complexant having coordination sites available, the alkali metal entrapped in the coordination sphere is positively charged, whereas the free alkali metal atom gains a negative charge. The only example of an alkalide where the alkali metal cation is not bound in a cavity is of Janus alkalide [6,10], where the alkali metal cation and anion are present on the open faces of a molecule. These structural variations have a significant impact on the nonlinear optical properties. For example, the *β*_o_ value of Li^+^(NH_3_)_4_M^−^ with a cage-like complexant (NH_3_)_4_ is about six times larger than that of the corresponding Li^+^(calix[4]pyrrole)M^-^ with cup-like complexant calix[4]-pyrrole [4]. Moreover, the nonlinear optical response of the Janus alkalide is much larger than other cage-like alkalides. Similarly, borates consisting of boron oxyanions are a fast-growing field of solid-state chemistry with well-established and important functionality [20]. In recent decades, freshly discovered borates gained the spotlight in research due to the great variety of their molecular structures (0D, 1D, 2D and 3D) [21,22].

Despite these advancements in the rational design of alkalides, one does not find any parallel rational design strategy for transition metal anions. The literature reveals a number of molecules/complexes where transition metals exist as anions. However, for transition metal anions, the difference in electropositivity of combining atoms is generally taken as the guiding principle. For example, platinum and gold atoms in Cs2Pt [23] and CsAu [24,25,26,27] bear a −2 and −1 charge, respectively. In these compounds, the transition metal atom acts as a reservoir to hold 12 electrons in 6s2 5d10 configuration for Pt^2−^ and Au^1−^. A similar situation is observed for semiconducting oxides containing 18 electron octahedral cations [PtIn_6_]^10+^ and [IrIn_6_]^9+^ [28,29]^.^ A quick glance at the structure of these cations reveals that the platinum and iridium in these complexes are in −2 and −3 oxidation states.

From the discussion above, one can clearly see that the transition metal anions are all covalent compounds where transition metal is covalently bound to some other electropositive atom (vide supra). Moreover, a design strategy of the simple coordination compound containing the transition metal anion is lacking. The only example reported in the literature for a coordination-based metalide is by Sun and co-workers, who have recently shown that the judicious placement of coinage metal and alkali metals on opposite faces of all-*cis*-1,2,3,4,5,6 hexfluorcyclohexane can generate metalides with a enhanced nonlinear optical response [30]. Moreover, density functional theory (DFT) calculations have also been extensively reported in the literature on the geometric, thermodynamic stability and electronic properties of heterofullerenes for nonlinear optical response [31,32].

With these targets, a new design strategy is required. If the structures of the alkalides is analyzed, one can clearly see that the alkali metal atom inside the cage gains a positive charge because the positive charge is balanced by an electron donation from electron-rich nitrogen or oxygen atoms. It is proposed here that if the entrapped alkali atom is surrounded by electropositive atom, then it may receive a negative charge without the assistance of any other compensating alkali metal cation. In this regard, the molecules of choice are inorganic fullerenes such as X_12_Y_12_ where X = Al or B and Y = N or P. These inorganic fullerenes have electropositive and electronegative atoms available. Although these contain electronegative atoms present in the sphere, they also have electropositive atoms. If the effect of electropositive atoms dominates over the electronegative atoms, then the encapsulation of transition metals in these fullerenes may provide an easy approach to generate metalides. The diversity of atoms present in these fullerenes may help in finding a suitable candidate in this regard.

These inorganic fullerenes can exist in a variety of sizes such as X_12_Y_12_, X_16_Y_16_, X_28_Y_28,_ etc. Theoretical studies illustrate that X_12_Y_12_ nanoclusters are more stable than higher analogues [33,34]. X_12_Y_12_ nanocages are the subject of extensive exploration in the recent past for their application in sensors [35,36,37], storage [38,39], batteries and nonlinear optical materials [40,41,42,43,44]. Several studies reveal that the doping of alkali metal, whether exohedral or substitutional, increases the hyperpolarizability of the nanocage [40,41,43,44,45,46,47,48,49]. Xuri Huang and coworkers have shown that the exohedral doping of alkali metal significantly enhances the hyperpolarizability of Al_12_N_12_ nanocages. The authors argued that diffuse excess electrons are responsible for boosting the hyperpolarizability. Similarly, Maria et al. have shown that substitutional doping of alkali metal for X or Y increases the hyperpolarizability by two to three orders of magnitude [40,41,42]. However, this increase is less when compared with those for exohedral doping. Endohedral doping of alkali metals in these fullerenes is also reported for their nonlinear optical response. However, the alkalide characteristic of these EMFs is not discussed. The focus of the current study is to examine the metalide character of transition-metal-encapsulated fullerenes. Encapsulation of the metal atom imparts some unique characteristics to the nanocages, which are non-existent in pure nanocages. Endohedral doping of nanocages have found possible applications in many areas of scientific research including renewable energy, photonics and optoelectronics [50,51,52]. Moreover, the hyperpolarizability of EMF is discussed for the first time. A complete systematic spin-polarized DFT study is carried out.

## 2. Methodology

All calculations are performed with Gaussian 09 [53], whereas the visualization of the results was achieved through GaussView [54]. The geometry of all structures were optimized without any symmetry constraints at B3LYP/6-31G(d,p). It has been shown by us and others that the geometries of these nanocages are predicted reliably with the hybrid B3LYP method [35,55,56,57,58]. Moreover, the geometries are not affected to any appreciable extent when dispersion-corrected methods (ωB97XD and M05-2X) are applied. For transition metals, the spin-polarized DFT study was carried out in which the four lowest possible spin states are evaluated. The geometries of these endohedral metallofullerenes were confirmed at true minima through frequency analysis (lack of any imaginary frequency). The charge analysis was performed through natural bonding orbital (NBO) approach to realize the metalide nature of these complexes. S^2^ values for all complexes are evaluated to realize any spin contamination. The binding energies are calculated for these complexes in order to evaluate their stabilities. The binding energies are calculated through the following equation.

Eb = E_M@X12Y12_ − (E_X12Y12_ + E_M_) (1)

where E_X12Y12_, E_M_ and E_M@X12Y12_ are the total energies of the undoped X_12_Y_12_ molecule, the metal atom M and the corresponding doped system M@X_12_Y_12_, respectively. The bond order is also calculated through NBO approach for all these complexes.

Since polarizability is a measure of the distortion of the electronic cloud under the influence of an applied electric field, the relationship between the energy of a system in a weak and homogenous electric field is as follows.

E = E^o^ − *µ*_α_F_α_ − 0.5α_α*β*_F_α_F_*β*_ − 1/6*β*_α*β*γ_F_α_F_*β*_F_γ_ − … (2)

where F_α_ is the electric field component along the α direction and E^o^ is the molecular total energy without electric field. *µ*_α_, α_α*β*_, and *β*_α*β*γ_ are the dipole, the polarizability and the first hyperpolarizability, respectively.

Hyperpolarizability is calculated at the long-range-corrected CAM-B3LYP and LC-BLYP methods because the conventional DFT methods overestimate the hyperpolarizability due to incorrect electric field dependence provided by exchange correlation functional. The CAM-B3LYP method provides long range correction through Coulomb attenuation method, and it is superior for calculating NLO properties.

The mean polarizability and hyperpolarizability are described as

α = 1/3(α_xx_ + α_yy_ + α_zz_) (3)

*β*_0_ = (*β*_x_^2^ + *β*_y_^2^ + *β*_z_^2^)^1/2^ (4)

*β_vec_*, which is more relevant to a measurable quantity, is the projection of *β* on the dipole moment vector and is given by the following equation [59]:

(5) $$\beta_{vec}=\frac{\sum \mu i\beta i\;}{\left|\;\mu \right|}$$

where *µ_i_*, *β_i_* and |*µ*| are the dipole moments along the *i* direction, polarizability along the *i* direction and the total dipole moment, respectively.

UV-Vis calculations are performed through the time-dependent DFT approach (TD-DFT) at LC-BLYP method (TD-LC-BYLP) along with 6-31+G(d) basis set. A total of twenty states were computed with the 50:50 singlet triplet. These computations were performed with unrestricted formalism. The well-parametrized Bethe–Salpeter Green’s function approach has an advantage that it is parameter free and has been shown to provide reliable results in a large variety of organic or inorganic systems, with an insulating or metallic character, in extended (bulk) system or finite (reduced) size systems [60,61,62,63,64]. However, the Green’s function formalism cannot be considered to provide higher accuracy than DFT/TDDFT simulations [65].

A two-level model proposed by Oudar and Chemla [66] is also applied to understand the contributing factors for hyperpolarizability.

## 3. Results and Discussion

### 3.1. Spin State Analysis

For transition-metal-doped systems, it is better to discuss the spin states prior to discussing the optimized geometries. As presented above, the geometries of transition-metal-doped nanocages are optimized for the four lowest possible spin states. These include doublet, quartet, sextet and octet for Sc, V, Mn, Co and Cu. The spin states studied for Ti, Cr, Fe, Ni and Zn are singlet, triplet, quintet and septet. The results (Table 1) do not deliver any obvious trends at first glance because the most stable spin states are changing with the nature of the nanocage, except for late transition metals Ni, Cu and Zn, where stable spin states are singlet, doublet and singlet, respectively, regardless of the nature of the nanocage. The endohedral cobalt complexes **endo-Co@X_12_Y_12_** are not very different where the most stable spin state is doublet for all nanocages, except for Al_12_N_12_ (**endo-Co@Al_12_N_12_**), where the most stable spin state is quartet. There are some other generalizations available for the data presented in Table 1. For example, transition metals prefer a higher spin in Al nanocages (Al_12_N_12_ and Al_12_P_12_). Additionally, the most stable spin states of Ti, V, Cr, Mn and Fe in Al_12_N_12_ nanocage are quintet, quartet, septet, sextet and quintet, respectively. The stable spin states of Ti, V, Cr, Mn and Fe in Al_12_P_12_ are quintet, sextet, septet, sextet and quintet, respectively.

The results in Table 1 also give information about the energy differences of higher spin states from the lowest energy spin state. The energy difference between the lowest energy spin state and the next spin state is significant in all cases except for **endo-Co@Al_12_N_12_** and **endo-Co@Al_12_P_12,_** where the differences are 0.92 and 0.43 kcal mol^−1^, respectively. For Al_12_N_12_, small differences between the two lowest spin states are also observed for **endo-Fe@Al_12_N_12_** and **endo-Ni@Al_12_N_12_**: 1.41 and 0.44 kcal mol^−1^, respectively. The highest difference in energy between the two states is observed for **endo-Cu@X_12_Y_12_** (for all systems). These values are 44.17 and 45.35 kcal mol^−1^ for **endo-Cu@Al_12_N_12_** and **endo-Cu@Al_12_P_12_**, respectively.

The symmetry group of both optimized nanocage **Al_12_N_12_** and **Al_12_P_12_** is *T_h_*. In the case of **endo-TM@Al_12_N_12,_** the *T_h_* is retained for Cr and Zn metal, whereas for Mn the symmetry observed is *C_3,_* while the rest of the complexes show *C_1_* symmetry. Similarly, in case of endo-**TM@Al_12_P_12_**, the *T_h_* is retained for Mn and Zn metal, whereas *C_3_* symmetry is observed for Ti and Ni. Moreover, *C_2V_* symmetry in obtained for Fe, while all the rest of the complexes exhibit *C_1_* symmetry. Even when the symmetry is not *C_1_* for the most stable spin state (as mentioned above), the higher spin states show *C_1_* state in these cases, probably due to the distortion faced in the higher spin state.

### 3.2. Optimized Geometries

**Figure 1:**
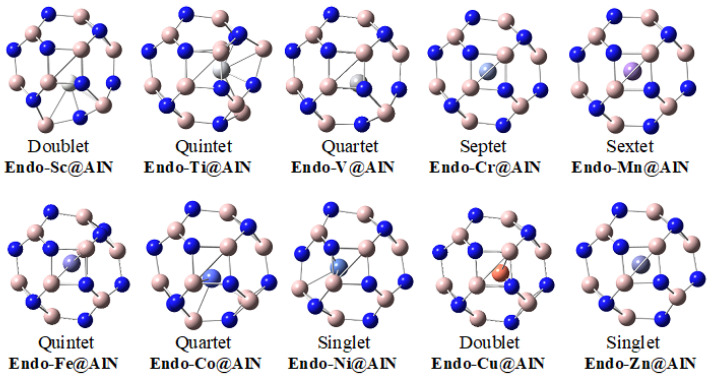
Optimized geometries and the most stable spin states of **endo-TM@Al_12_N_12_** complexes.

The optimized geometries of transition-metal-doped Al_12_N_12_ nanocages are shown in Figure 1. Sideview images are shown for the proper position of transition metal in the nanocage. The optimized geometries of all structures (Figure 1) indicate that most of the transition metals are not present at the center of the nanocages except for certain middle transition metal atoms (Cr, Mn and Fe) and Zn. These results are somewhat perplexing because early transition metal atoms are more electron deficient, and they tend to bind with high coordination number in order to overcome their electron deficiency. The central orientation of Zn in Al_12_N_12_ nanocage may be attributed to the lack of a binding tendency in Zn due to the completely filled d orbital, as it is also evident from binding energies analysis (vide infra). The change in the bonding orientation of different transition metals in the nanocage has a strong impact on the charges of transition metals (see NBO section).

**Figure 2:**
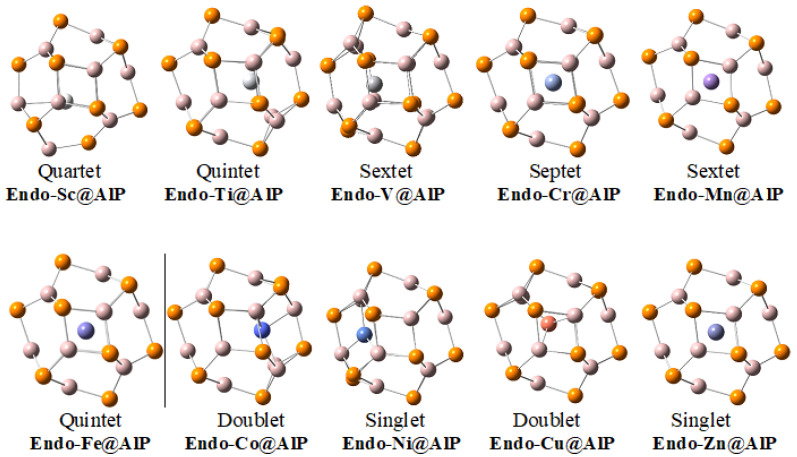
Optimized geometries and the most stable spin states of **endo-TM@Al_12_P_12_** complexes.

An identical situation is observed for **endo-TM@Al_12_P_12_** complexes, where early transition metals Sc, Ti and V tend to sit on a side of the nanocage, but Cr, Mn and Fe reside almost at the center of the nanocage (Figure 2). Co, Ni and Cu are also oriented towards a side of the nanocage, but Zinc is again present at the center of the nanocage. It is worth mentioning that the complexes where transition metal is not present at the center are not similar to each other. In some cases, the transition metal is facing towards a hexagon of the nanocage, whereas, in others, it is facing a tetragon. Among all complexes, endo-Zn@X_12_Y_12_ complexes only contain the metal atom at the center of the nanocages for all inorganic fullerenes, which mainly attributed to lack of any tendency for coordination due to completely filled d orbital.

### 3.3. Binding Energies

The binding energies of transition metals with the nanocages are analyzed (Table 2). Obviously, no trend could be traced in the interaction energies. The interaction of early transition metals with the Al_12_N_12_ is an exothermic process. The Gibbs free energies of the interaction of early transition metals show an odd–even effect. The interaction energy of Sc with Al_12_N_12_ is −12.56 kcal mol^−1^ increases to −41.72 for **endo-Ti@Al_12_N_12,_** but again shows decrease and increase to −8.78 and −13.95 kcal mol^−1^ for **endo-Vi@Al_12_N_12_** and **endo-Cr@Al_12_N_12_**, respectively. The Gibbs free energies are positive for **endo-Mn@Al_12_N_12_** and **endo-Fe@Al_12_N_12_**. The Gibbs free energies are 38.83 and 9.79 kcal mol^−1^ for **endo-Mn@Al_12_N_12_** and **endo-Fe@Al_12_N_12_**, respectively. These are the complexes where transition metal resides at the center of the nanocage (vide supra). From the results, it is obvious that central orientation of transition metal in Al_12_N_12_ is because of the weak interaction of the metal with the nanocage, except for **endo-Cr@Al_12_N_12,_** where the interaction is exothermic despite the fact that the metal resides in the center of the nanocage. The geometric parameters revealed that Zn in **endo-Zn@Al_12_N_12_** is also oriented in the center of the nanocage. The interaction energy is also positive for this complex which supports the above-mentioned notion. For these metal-encapsulated nanocages, two factors are operational: (a) attractive force between metal and nanocage and (b) repulsive interaction between nanocage and metal due to steric interaction. For weakly coordinating metals such as Mn and Zn, the repulsive interactions dominate over weak attractive forces, which results in overall endergonic process. For Al_12_N_12_, the strongest interaction is observed when nickel atom is encapsulated in the nanocage (−77.84 kcal mol^−1^). The binding of Cu with Al_12_N_12_ is also strongly exergonic by −38.17 kcal mol^−1^.

Al_12_P_12_ nanocage is larger in size; therefore, the repulsive interaction between nanocage and metal atoms is expected to be negligible. Analysis of the interaction energies reveals that the interaction of all transition metals with Al_12_P_12_ nanocage is favorable. There are some common features between the **endo-TM@Al_12_N_12_** and **endo-TM@Al_12_P_12_** series. For example, the interaction energies of **endo-Mn@Al_12_P_12_**, **endo-Fe@Al_12_P_12_** and **endo-Zn@Al_12_P_12_** are lower than other complexes, but fortunately, these interaction energies are exothermic for Al_12_P_12_ when compared with those of Al_12_N_12_ complexes. The interaction energies in **endo-Mn@Al_12_P_12_**, **endo-Fe@Al_12_P_12_** and **endo-Zn@Al_12_P_12_** are −7.83, −19.14 and −17.01 kcal mol^−1^, respectively. The exergonic energies of the interaction are mainly because of the lack of repulsive interactions from the nanocage with the encapsulated transition metals. In the absence of repulsive forces, attractive forces are only playing their role and the interaction energies are negative. Moreover, the interaction energy of Ni with Al_12_P_12_ (**endo-Ni@Al_12_P_12_**) is the highest in the series, a characteristic common with Al_12_N_12_ nanocage. Despite these similarities, a marked difference is also observed. The odd–even oscillation of interaction energies of early transition metals with Al_12_N_12_ nanocages is not observed for Al_12_P_12_ nanocage. The interaction energies of **endo-Sc@Al_12_P_12_**, **endo-Ti@Al_12_P_12_**, **endo-V@Al_12_P_12_** and **endo-Cr@Al_12_P_12_** are −58.36, −56.00, −55.83 −53.10 kcal mol^−1^, respectively. For Al_12_P_12_, the highest interaction energy is observed for **endo-Ni@Al_12_P_12,_** followed by **endo-Co@Al_12_P_12_** and **endo-Cu@Al_12_P_12_**.

### 3.4. Expansion of the Nanocage

It was of quite interest to see whether encapsulation causes expansion of the nanocage. The expansion of the nanocage is calculated by diameter of the nanocages between two face-to-face hexagons (Figure 1) before and after encapsulation. Analysis of the data reveals that encapsulation of transition metal causes expansion of the nanocages; however, an irregular trend is observed. For **endo-TM@Al_12_N_12_** complexes, the maximum increase in the diameter is observed for Sc encapsulation (0.157 Å), and it decreases slightly for **endo-Ti@Al_12_N_12_** (0.154 Å). The diameters of **endo-Sc@Al_12_N_12_** and **endo-Ti@Al_12_N_12_** are 4.597 and 4.594 Å, respectively, compared to 4.44 for the bare nanocage, and the maximum increase in the diameter of these nanocage (**endo-Sc@Al_12_N_12_** and **endo-Ti@Al_12_N_12_**) is attributed to their larger size. The expansion of the nanocage decreases with the increase in the atomic number of the transition metal up to **endo-Ni@Al_12_N_12,_** except for **endo-Mn@Al_12_N_12,_** where slightly pronounced increase in the expansion is observed. This irregular pronounced expansion of **endo-Mn@Al_12_N_12_** is attributed to half-filled nature of Mn, which resists in coordination with other atoms. The data reveal that the Al_12_N_12_ nanocage is not affected by nickel encapsulation which indicates that the size of the nickel atom is ideal to fit in the cavity of the nanocage. After **endo-Ni@Al_12_N_12_**, the ring expansion increases again. The diameters of **endo-Cu@Al_12_N_12_** and **endo-Zn@Al_12_N_12_** are 4.480 and 4.503, respectively, compared to 4.44 for the bare nanocage.

Quite similar to Al_12_N_12_ nanocage, an irregular trend is also seen for endo-TM@Al_12_P_12_. However, there are certain common features. For example, the maximum expansion (0.082) is seen for **endo-Sc@Al_12_P_12_**. The diameter of **endo-Sc@Al_12_P_12_** is 5.532 Å compared to 5.45 Å for the bare Al_12_P_12_ nanocage. Another common characteristic between the two nanocages is the irregular increase in the diameter for **endo-Zn@Al_12_N_12_** and **endo-Mn@Al_12_N_12,_** which is again attributed to lack of any tendency for coordination due to completely filled and half-filled d orbitals. Surprisingly, the Al_12_P_12_ nanocage shrinks slightly to accommodate copper atoms. The diameter of **endo-Cu@Al_12_P_12_** is 5.44 compared to 5.45 for the bare nanocage. Similar shrinkage of the nanocage is also seen for **endo-Co@Al_12_P_12_**, **endo-V@Al_12_P_12_** and **endo-Ni@Al_12_P_12_**. The maximum shrinkage in the diameter of the nanocage is seen for **endo-Ni@Al_12_P_12_**. The data reveal that the Al_12_P_12_ nanocage is ideal for copper atom to fit in.

### 3.5. Charge Analysis and Dipole Moment

Next, attention was paid to a very important question: what is the charge of encapsulated metal in these nanocages? Since transition metal offers more diversity in their bonding, their behavior towards charges is worth studying. It was initially thought that placing a transition metal inside inorganic fullerenes will lead to the formation of metalides. Charge analysis reveals that the charge of encapsulated transition metal in the nanocage is strongly dependent on the nature of transition metal and the nanocage. For **endo-TM@Al_12_N_12_**, the general trend is a decrease in positive charge (or increase in the negative charge) while moving from Sc to Zn. (A few exceptions do exist.) The early transition metals are electron deficient; they tend to receive positive charge when encapsulated in the nanocage. The late transition metals are relatively less electron deficient, and they gain negative charge upon encapsulation. The positive charge of early transition metals decreases as the atomic number increases. The NBO charges on Sc, Ti, V and Cr in their respective complexes of Al_12_N_12_ **endo-Sc@Al_12_N_12_**, **endo-Ti@Al_12_N_12_**, **endo-V@Al_12_N_12_** and **endo-Cr@Al_12_N_12_** are 0.53, 0.41, 0.37 and −0.04, respectively. It is interesting to note that the positive charges on these metal atoms are not as pronounced as expected for early transition metals. Cr in **endo-Cr@Al_12_N_12_** is negatively charged, although at a very small number. These findings are quite interesting and justify the proposed notion. The negative (or lower positive) charges on the transition metals in these complexes are mainly due to the presence of a large numbers of aluminum atoms in the vicinity, which take most of the positive charge. Moreover, the aluminum atoms in these inorganic fullerenes are bound to interact with transition metals at distances much shorter than their van der Waals radii, as compared to electronegative nitrogen atoms. Therefore, the effect of the aluminum atom on the charges of transition metals are more pronounced than the nitrogen atoms. The anionic character of the transition metal is also noticed for the complexes of the late transition metals. The charges on Co, Ni, Cu and Zn in **endo-Co@Al_12_N_12_**, **endo-Ni@Al_12_N_12_**, **endo-Cu@Al_12_N_12_** and **endo-Zn@Al_12_N_12_** are −0.11, −0.27, −0.27 and −0.17, respectively. The charges increase from Co to Cu, and then a slight decrease is observed for Zn. The decrease in anionic character for Zn may be attributed to the weak bonding of Zn with the nanocage. The strongest anionic character is seen for Ni and Cu (0.27 charge each) in their respective complexes. Although the charges on the transition metals are not very strong negatives, at least they provide a new way to generate negative charges on transition metals by encapsulating in inorganic fullerenes.

Interestingly, the negative character of the transition metals is pronounced in Al_12_P_12_ nanocages. All encapsulated transition metals except Sc, Ti and V show a negative charge. Sc, Ti and V are positively charged in their complexes; however, their charges are lesser than their corresponding charges in Al_12_N_12_ nanocages. For example, the charge of Sc in **endo-Sc@Al_12_P_12_** is 0.36 compared to 0.53 for **endo-Sc@Al_12_N_12_**. For **endo-Cr@Al_12_P_12_**, **endo-Fe@Al_12_P_12_** and **endo-Co@Al_12_P_12_**, the negative charges are quite low (in the range of −0.02 to −0.06. The highest negative charge of −0.41 is calculated for **endo-Zn@Al_12_P_12_**. For Al nanocages, the phosphides are better for metalide character, whereas among B nanocages, nitrides are better. It can be rationalized on soft and hard acid base (SHAB) concept.

Another characteristic closely associated with the charges is the dipole moment. The dipole moments of **endo-TM@X_12_Y_12_** are also analyzed. The dipole moments do not show any regular trend, quite similar to the charges. However, one thing is quite obvious, which is that the complexes where transition metal is present at the center of the nanocage (such as **endo-Cr@Al_12_N_12_**, **endo-Mn@Al_12_N_12_** and **endo-Zn@Al_12_N_12_**) do not show any appreciable dipole moments. Dipole moments of these complexes are in the range of 0.05–0.07 D. This is quite logical because the dipole moment depends on the charges as well as separation between them. The transition metal atoms in these complexes bear low charges. Moreover, individual dipole moment vectors are cancelled out due to the centrosymmetric nature of the complex. The highest dipole moment (6.11 D) is observed for **endo-Sc@Al_12_N_12_** nanocages followed by 4.64 D for **endo-Co@Al_12_N_12_**. The dipole moments of **endo-Ti@Al_12_N_12_** and **endo-V@Al_12_N_12_** (3.14–3.15 D) are also significantly high. From the results, one more obvious fact is that the magnitude of the dipole moment is not related to the charge only. For example, the Ni and Cu both have similar charges (−0.27) in **endo-Ni@Al_12_N_12_** and **endo-Cu@Al_12_N_12_**, respectively, but the dipole moments are remarkably different. The dipole moments of **endo-Ni@Al_12_N_12_** and **endo-Cu@Al_12_N_12_** are 0.24 and 4.64 D, respectively. This is mainly due to the separation of charges in the nanocage between Al and N atoms. For **endo-Cu@Al_12_N_12_**, the charge on the nitrogen and aluminum atoms of the nanocages is higher compared to those in **endo-Ni@Al_12_N_12_** (see Table 3). The symmetry-group-related discussion for both optimized nanocage **Al_12_N_12_** and **Al_12_P_12_** is delineated in the spin-state analysis (Section 3.1).

**Figure 3:**
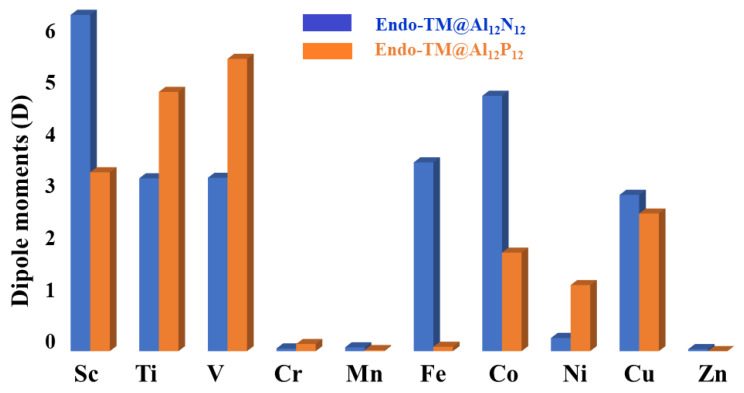
Illustration of dipole moments of **endo-TM@X_12_Y_12_** fullerenes.

As shown in Table 3 and Figure 3, the dipole moments of **endo-TM@Al_12_P_12_** follow a trend similar to those of **endo-TM@Al_12_N_12,_** where early transition-metals-based complexes show higher dipole moments, whereas lower values are seen for complexes of middle transition metals **endo-TM@Al_12_P_12_** (M = Cr, Mn and Fe). The dipole moments of these complexes (**endo-TM@Al_12_P_12_** (M = Cr, Mn and Fe)) and **endo-Zn@Al_12_P_12_** are low due to the same reason as seen above for **endo-TM@Al_12_N_12_**; the transition metals are present exactly at the center of the fullerenes, and individual dipole moments are cancelled out. In this series, the highest dipole moment is seen for **endo-V@Al_12_P_12_**.

### 3.6. Frontier Molecular Orbital Analysis

Transition metals offer much diversity for their charges and provide small incremental steps for changes in properties with changes in atomic number and radius. Since some of the metals show metalide character while others do not, their frontier orbital analysis will deliver useful information. The highest occupied molecular orbitals of all transition metal doped nanocages are analyzed. For **endo-TM@Al_12_N_12_**, the early transition metals (Sc-V) show positive charge in Al_12_N_12_ nanocage, whereas late transition metals (Ni, Cu, Zn) show metalide character.

**Figure 4:**
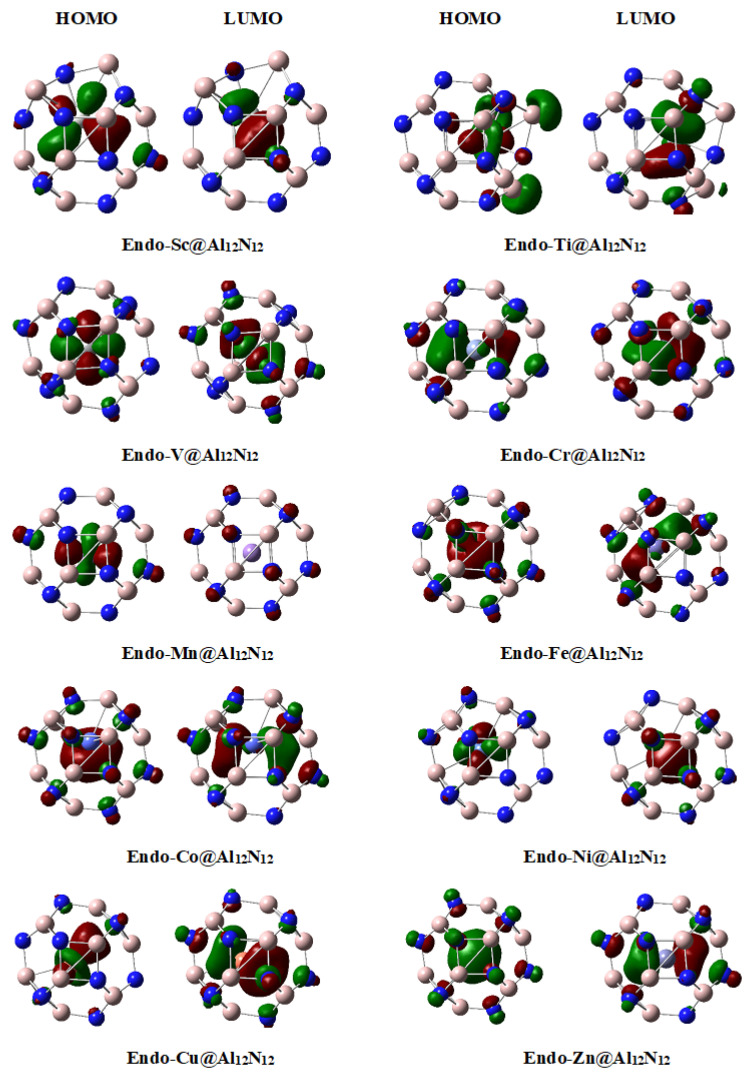
Frontier molecular orbitals of **endo-TM@Al_12_N_12_** complexes plotted at an isovalue of 0.045.

The HOMOs of these complexes show quite interesting chemistry lying underneath. For **endo-Sc@Al_12_N_12_**, **endo-Ti@Al_12_N_12_** and **endo-V@Al_12_N_12_**, the HOMOs are localized mainly on the transition metal (Figure 4). The orbitals plots show that the highest occupied molecular orbitals in these complexes are d orbitals of transition metals. The presence of HOMO on d orbitals indicates that these complexes are excess electron systems. These d orbital electrons are pushed out under the influence of lone pairs of nitrogen in Al_12_N_12_ nanocage. These excess electrons are well known to impart large nonlinear optical responses [67,68,69,70,71,72]. We and others have shown previously that such excess electrons on alkali and transition metals can lead to significantly large nonlinear optical response in these systems, particularly for Al_12_N_12_ cluster.

The situation is quite interesting for **endo-Cr@Al_12_N_12,_** where HOMO has density in empty space in close proximity of the transition metal, which indicates that this complex is an electride. The NBO charge analysis reveals that transition metal in this case (Cr) has a slight negative charge (metalide), and one should expect HOMO density on transition metal for metalides. Since the HOMO density of this electride is present in empty space near the transition metal, it is easy to understand that the small negative charge in the transition metal is due to the electride characteristic where some part of the density is on the metal. Therefore, the **endo-Cr@Al_12_N_12_** complex is at the borderline between the metalide and the electride; however, the latter effect is dominating. A similar borderline (between metalide and electride) situation is also seen for **endo-Co@Al_12_N_12,_** where the HOMO indicates electride character; however, the NBO charges show some metalide characteristics. A difference between **endo-Cr@Al_12_N_12_** and **endo-Co@Al_12_N_12_** is the positioning of the density in HOMO. In the former, the density is slightly away from the metal center, but in the latter case, it is much closer to the metal center.

**Endo-Ni@Al_12_N_12_** and **endo-Cu@Al_12_N_12_** complexes are true metalides because in these cases, the HOMOs are centered on the transition metals, which when combined with NBO charge analysis (negative charge on metal) confirms the metalide character. The HOMO is a d_xy_ type orbital in **endo-Ni@Al_12_N_12,_** whereas it is a d_z_^2^ orbital in **endo-Cu@Al_12_N_12_**. **Endo-Zn@Al_12_N_12_** complex is also a metalide, but the HOMO in this complex is not present on a d orbital; rather, it is an s orbital on Zn. In summary, **endo-TM@Al_12_N_12_** complexes offer quite a diversity where the first three members have excessive electron phenomenon (without any metalide or electride characteristic), while the next three members are at the borderline between metalide and electride. The last four members of the series are true metalides.

**Figure 5:**
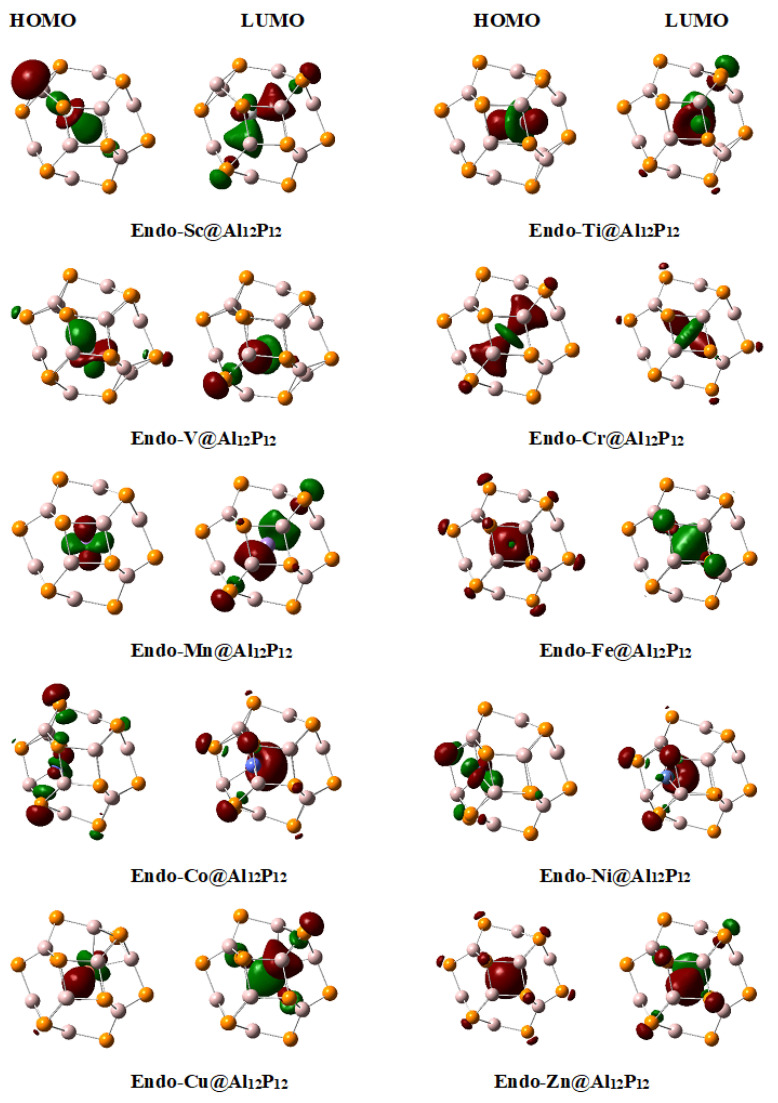
Frontier molecular orbitals of **endo-TM@Al_12_P_12_** complexes plotted at isovalue of 0.045.

Next, the frontier orbitals of **endo-TM@Al_12_P_12_** complexes are analyzed. First, three members of the series (**endo-Sc@Al_12_P_12_**, **endo-Ti@Al_12_P_12_** and **endo-V@Al_12_P_12_**) show behavior very similar to those of their corresponding **endo-TM@Al_12_N_12_** complexes (Figure 5); these are simply excess electron systems which are characterized by the presence of HOMO on transition metal (positive charge on metal from NBO analysis). Although the HOMOs are present on transition metals in these complexes, the d orbital involved in HOMO (d_z_^2^) is different than those of the corresponding **endo-TM@Al_12_N_12_** complexes where d_xy_ type orbital is involved in HOMOs. Although the first three members in both series show excess electron nature, the complexes from middle transition metals show quite opposite behavior. **Endo-TM@Al_12_P_12_** (TM = Cr-Zn) are all metalides except **endo-Fe@Al_12_P_12,_** which shows electride characteristics. An interesting feature of all these **endo-TM@Al_12_P_12_** (TM = Cr-Zn) complexes is the involvement of d_z_^2^ orbital in HOMOs, except **endo-Zn@Al_12_P_12,_** where s orbital is involved.

Next, the energies of the frontier molecular orbitals are analyzed (Table 4). For **endo-TM@Al_12_N_12_**, the HOMO-LUMO gaps are reduced significantly by encapsulation of transition metals when compared with the bare Al_12_N_12_ nanocage (3.93 eV). The HOMO-LUMO gap (E_H-L_) for **endo-Sc@Al_12_N_12_** is 1.67 eV, which shows a reduction of 57.6% compared to that of the undoped system. An odd–even effect on the E_H-L_ is seen in this series. However, the lowest E_H-L_ is seen for **endo-Fe@Al_12_N_12,_** where the E_H-L_ is 1.55 eV and the percent change in E_H-L_ is about 60.5. The next lowest E_H-L_ is seen for **endo-V@Al_12_N_12_**. The highest E_H-L_ in this series is seen for **endo-Zn@Al_12_N_12_**. Doping of transition metals in Al_12_P_12_ nanocages also reduces the E_H-L;_ however, the effect is not as much pronounced as for the **endo-TM@Al_12_N_12_**. For **endo-TM@Al_12_P_12_**, the lowest E_H-L_ is seen for **endo-Sc@Al_12_P_12_**, where the E_H-L_ is reduced to 1.53 eV compared to 3.39 eV for the bare nanocage. The next pronounced change in E_H-L_ is seen for **endo-Co@Al_12_P_12,_** where the E_H-L_ is 1.72 eV (49.4% decrease). For **endo-TM@Al_12_P_12_**, an odd–even effect is seen for E_H-L,_ where the odd electron-containing species havelower E_H-L,_ whereas the even electron-containing species have higher E_H-L,_ except **endo-Mn@Al_12_P_12_**. The odd behavior of **endo-Mn@Al_12_P_12_** may be attributed to the high stability of the Mn because of half-filled d orbital. A similar but more pronounced effect is seen for **endo-Zn@Al_12_P_12,_** where E_H-L_ is, in fact, increased to 3.62 eV (quite contrary to the rest of the series). The E_H-L_ analysis reveals that the gaps are reduced significantly by doping. This reduction in the energy gap is quite crucial for their potential application in optoelectronic devices because the large gaps of the bare nanocages hinder their applications. It is also worth mentioning that the E_H-L_ are reduced, though they are not reduced too much to the extent that these systems are kinetically unstable. The E_H-L_ are in reasonable range for enough electronic stability. For most of the optical applications’ higher transmittance, the visible region is of great interest. The NLO materials having an energy gap in the visible region finds application in material devices such as sensors, light emitters, photonics, optical communication and many other newly emerging applications [73,74,75]. Overall, the higher transmission in the visible range enables these NLO materials to be promising candidates for optoelectronic applications.

### 3.7. Nonlinear Optical Response

The literature reveals that alkalides are the materials with a high nonlinear optical response. It is worth investigating whether these “metalides” or other transition-metal-doped endohedral fullerenes have potential for nonlinear optical materials or not. The polarizability and first hyperpolarizability (nonlinear optical response) of the endohedral “metalides” **endo-TM@X_12_Y_12_** is discussed in Table 5 and compared with those of the endohedral alkalides and others from the literature.

The polarizability of bare nanocages is 285 and 598 a.u. for Al_12_N_12_ and Al_12_P_12_, respectively. For **endo-TM@Al_12_N_12_,** an interesting odd–even oscillation in polarizability values is observed. For example, the polarizability of **endo-Sc@Al_12_N_12_** is 486 au, and it decreases to 341 au for **endo-Ti@Al_12_N_12,_** but again increases to 522 for **endo-V@Al_12_N_12_**. The highest polarizability in this series is seen for **endo-Mn@Al_12_N_12,_** whereas the lowest is for **endo-Ni@Al_12_N_12_**. The odd–even oscillation seen for **endo-TM@Al_12_N_12_** is not observed for other complexes.

The bare nanocages (Al_12_N_12_, Al_12_P_12_) are centrosymmetric and therefore have zero hyperpolarizability. Introduction of a transition metal changes the hyperpolarizability significantly. From the results, it appears that the hyperpolarizability of **endo-TM@X_12_Y_12_** complexes is independent of the nature of the species whether the complex is a transition metalide or doped transition metal with a positive charge. For example, for **endo-TM@Al_12_N_12_**, the hyperpolarizability follows an odd–even effect, quite similar to polarizability. In this odd–even effect, the highest hyperpolarizability is seen for **endo-V@Al_12_N_12_** (2.80 × 10^5^ au). The next highest hyperpolarizability is calculated for **endo-Sc@Al_12_N_12_**. The highest hyperpolarizability for any transition metalide is calculated for **endo-Co@Al_12_N_12_** (1.17 × 10^4^). Many other complexes with metalide character such as **endo-Cr@Al_12_N_12_**, **endo-Ni@Al_12_N_12_**, **endo-Cu@Al_12_N_12_** and **endo-Zn@Al_12_N_12_** have relatively low hyperpolarizability values. Interestingly, all these species except **endo-Cu@Al_12_N_12_** have metals with an even number of electrons. The hyperpolarizability of **endo-Cu@Al_12_N_12_** is 2342 au. From the results for **endo-Cr@Al_12_N_12_**, it is clear that the hyperpolarizability of odd electron systems is higher, an effect similar to alkali metal doping. Alkali metals have an odd number of electrons, and they impart large nonlinear optical response to inorganic fullerenes upon doping. It is also worth mentioning that the systems where transition metal resides in the center of the nanocage have relatively lower hyperpolarizability (as expected from the trend). For example, the hyperpolarizability of **endo-Mn@Al_12_N_12_** is merely 1.1 × 10^3^ au, whereas it is 2.80 × 10^5^ au for **endo-V@Al_12_N_12_**.

The odd–even oscillating trend of hyperpolarizability is also seen for **endo-TM@Al_12_P_12_** to some extent. Moreover, the hyperpolarizability values of complexes in this series are lower than those of **endo-TM@Al_12_N_12_**. For example, the highest values of hyperpolarizability in this series are calculated for **endo-Sc@Al_12_P_12_** (3954 au), which is about an order of magnitude smaller than that of **endo-Sc@Al_12_N_12_** (1.76 × 10^4^ au). The hyperpolarizability drops to 2345 au for **endo-Ti@Al_12_P_12,_** but again increases to 2773 au for **endo-V@Al_12_P_12_** followed by another decrease to 403 au for **endo-Cr@Al_12_P_12._** In this series, the lowest value is calculated for **endo-Zn@Al_12_P_12,_** followed by **endo-Mn@Al_12_P_12_**. Quite similar to AlN nanocage, the only metalide with reasonably high hyperpolarizability is **endo-Co@Al_12_P_12_** (probably mainly due to odd–even effect). Quite similar to AlN nanocages, the early transition-metal-doped AlP nanocages have relatively high hyperpolarizability. Both of these complexes have transition metal either half-filled or completely filled. Moreover, the transition metals are present exactly at the center of the nanocage. Both of these factors are probably responsible for low hyperpolarizabilities of these complexes.

For comparison, the polarizability and hyperpolarizability of **endo-TM@X_12_Y_12_** complexes are compared with some other reported metalides. Table 6 reveals that the first static hyperpolarizabilities of the studied complexes designed in this investigation are very much comparable or greater relative to the complexes reported in the literature, especially in case of **endo-V@Al_12_N_12_**.

## 4. Conclusions

Electronic and nonlinear optical properties of endohedral metallofullerenes are studied through density functional theory calculations. The endohedral metallofullerenes contain transition metal encapsulated in inorganic fullerenes X_12_Y_12_ (X = Al & Y = N, P). The endohedral metallofullerenes (**endo-TM@X_12_Y_12_**) possess quite interesting geometric and electronic properties, which are the function of the nature of atom and the size of fullerene. Early and late transition metals except **endo-Zn@X_12_Y_12_** tend to sit at the side of the cavity, whereas the middle transition metal atoms reside at the center of the fullerenes. NBO charge and frontier molecular orbital analyses reveal that the **endo-TM@Al_12_N_12_** are true metalides when transition metals are Ni, Cu and Zn. Additionally, **endo-Cr@Al_12_N_12_** and **endo-Co@Al_12_N_12_** are at the borderline between metalide and electride with predominant electride characteristics. The other members of the series are excess electron systems, which offer interesting electronic and nonlinear optical properties. The diversity of nature possessed by **endo-TM@Al_12_N_12_** is not prevalent for other fullerenes. Additionally, **endo-TM@Al_12_P_12_** are true metalides when transition metals are (Cr-Zn). Among all complexes studied, the highest metalide character is seen for **endo-Zn@Al_12_P_12,_** where the charge on the metal reaches up to −0.41. HOMO-LUMO gaps (E_H-L_) are reduced significantly for these endohedral metallofullerenes. Many complexes show odd–even oscillating behavior for E_H-L_ and dipole moments. Odd electron species contain large dipole moments and small E_H-L,_ whereas even electron systems have the opposite behavior. Despite a decrease in E_H-L,_ these systems show high kinetic and thermodynamic stabilities. The encapsulation of transition metals is a highly exergonic process. For all series, nickel-doped complexes are the most stable thermodynamically. These **endo-TM@X_12_Y_12_** possess remarkable nonlinear optical response where the first hyperpolarizability reaches up to 2.79 × 10^5^ au for **endo-V@Al_12_N_12_**. With few exceptions, the polarizabilities of the complexes are inversely related to the HOMO-LUMO gaps (E_H-L_). This study helps in the comparative analysis of nonlinear optical response of electrides, metalides and other excess electron systems. In general, the nonlinear optical response of electrides is higher than metalides but lower than those of simple excess electron compounds. In summary, the interesting electronic and large nonlinear optical response of the studied metalides shows that these materials are promising candidates for optoelectronic applications.

## Figures and Tables

**Table 1:** Illustration of ordering and relative energies of different spin states for endo-TM@X_12_Y_12_ complexes. All relative energies for a metal complex are with respect to its most stable spin state at 0 kcal mol^−1^.

	Spin States	Sc	Ti	V	Cr	Mn	Fe	Co	Ni	Cu	Zn
AlN	Stable spin	Doublet (0) *C_1_*	Quintet (0) *C_1_*	Quartet (0) *C_1_*	Septet (0) *T_h_*	Sextet (0) *C_1_*	Quintet (0) *C_1_*	Quartet (0) *C_1_*	Singlet (0) *C_1_*	Doublet (0) *C_1_*	Singlet (0) *T_h_*
	2nd stable	Quartet (2.72) *C_1_*	Triplet (2.13) *C_1_*	Sextet (5.22) *C_1_*	Quintet (3.18) *T_h_*	Quartet (3.41) *C_1_*	Triplet (1.41) *C_1_*	Doublet (0.92) *C_1_*	Triplet (0.44) *C_1_*	Quartet (44.17) *C_1_*	Triplet (21.09) *C_s_*
	3rd stable	Sextet (57.0) *C_1_*	Singlet (9.65) *C_1_*	Doublet (14.46) *C_1_*	Triplet (11.46) *C_1_*	Octet (4.40) *C_1_*	Septet (17.18) *C_1_*	Sextet (23.13) *C_1_*	Quintet (12.32) *C_1_*	Sextet (83.66) *C_1_*	Quintet (76.46) *C_1_*
	4th stable	Octet (122.9) *C_1_*	Septet (66.64) *C_1_*	Octet (70.54) *C_1_*	Singlet (44.37) *C_1_*	Doublet (19.68) *C_1_*	Singlet (28.09) *C_1_*	Octet (78.48) *C_1_*	Septet *C_1_*	Octet (154.63) *C_1_*	Septet *C_1_*
AlP	Stable spin	Quartet (0) *C_1_*	Quintet (0) *C_3_*	Sextet (0) *C_1_*	Septet (0) *C_1_*	Sextet (0) *T_h_*	Quintet (0) *C_2v_*	Doublet (0) *C_1_*	Singlet (0) *C_3_*	Doublet (0) *C_1_*	Singlet (0) *T_h_*
	2nd stable	Doublet (4.38) *C_1_*	Triplet (4.82) *C_1_*	Quartet (8.93) *C_1_*	Quintet (11.89) *C_1_*	Quartet (19.64) *C_1_*	Triplet (6.74) *C_1_*	Quartet (0.43) *C_1_*	Triplet (12.76) *C_1_*	Quartet (45.35) *C_1_*	Triplet (41.78) *C_1_*
	3rd stable	Sextet (43.88) *C_1_*	Singlet (9.58) *C_1_*	Doublet (12.55) *C_1_*	Triplet (36.26) *C_1_*	Octet (26.89) *C_1_*	Septet (13.70) *C_1_*	Sextet (29.09) *C_1_*	Quintet (55.88) *C_1_*	Sextet (90.70) *C_1_*	Quintet (89.62) *C_1_*
	4th stable	Octet (92.19) *C_1_*	Septet (47.18) *C_s_*	Octet (38.12) *C_1_*	Singlet (61.57) *C_1_*	Doublet (43.58) *C_1_*	Singlet (41.41) *C_1_*	Octet (78.85) *C_1_*	Septet (112.37) *C_1_*	Octet (144.85) *C_1_*	Septet (132.56) *C_3_*

**Table 2:** Zero point corrected and Gibbs free binding energies (kcal mol^−1^) of transition metals with X_12_Y_12_ nanocages.

Transition Metal	Nanocage			
	Al_12_N_12_		Al_12_P_12_	
	ZPE	Gibbs	ZPE	Gibbs
Sc	−20.72	−12.56	−64.47	−58.36
Ti	−49.68	−41.72	−62.63	−56.00
V	−16.74	−8.78	−62.74	−55.83
Cr	−20.93	−13.95	−58.07	−53.10
Mn	30.68	38.83	−14.42	−7.83
Fe	2.00	9.79	−23.84	−19.14
Co	−12.10	−4.45	−81.03	−73.76
Ni	−85.57	−77.84	−135.88	−127.54
Cu	−44.66	−38.17	−69.07	−61.24
Zn	21.35	28.11	−24.34	−17.01

**Table 3:** NBO Charges, diameters (in Å), change in diameters (in Å), dipole moments (in Debye), bond order, orientation of metal and symmetry of **endo-TM@X_12_Y_12_**.

Nanocage	Property	Transition Metals										
		Sc	Ti	V	Cr	Mn	Fe	Co	Ni	Cu	Zn	
Al_12_N_12_	Charge	0.53	0.41	0.37	−0.04	0.17	0.07	−0.11	−0.27	−0.27	−0.17	-
	Diameter	4.597	4.594	4.511	4.517	4.527	4.50	4.492	4.430	4.480	4.503	4.440
	Change	0.157	0.154	0.071	0.077	0.087	0.06	0.052	−0.01	0.04	0.063	
	Dipole Moment	6.11	3.14	3.15	0.05	0.07	3.43	4.64	0.24	2.84	0.037	0.0
	Bond order	4.27	4.39	3.31	2.13	2.57	2.60	2.62	3.35	2.56	2.48	-
	Symmetry	*C_1_*	*C_1_*	*C_1_*	*T_h_*	*C_3_*	*C_1_*	*C_1_*	*C_1_*	*C_1_*	*T_h_*	*T_h_*
	Orientation	Side	Side	Side	Center	Center	Side	Side	Side	Side	Center	
Al_12_P_12_	Charge	0.36	0.14	0.16	−0.02	−0.06	−0.12	−0.03	−0.17	−0.2	−0.41	-
	Diameter	5.532	5.460	5.423	5.490	5.504	5.490	5.428	5.397	5.442	5.500	5.45
	Change	0.082	0.010	−0.027	0.040	0.054	0.04	−0.022	−0.053	−0.008	0.05	
	Dipole Moment	3.25	4.71	5.31	0.13	0.02	0.08	1.79	1.20	2.50	0.00	0.0
	Bond order	3.22	2.80	2.76	2.55	2.91	3.09	2.91	3.50	2.87	3.00	-
	Symmetry	*C_1_*	*C_3_*	*C_1_*	*C_1_*	*T_h_*	*C_2V_*	*C_1_*	*C_3_*	*C_1_*	*T_h_*	*T_h_*
	Orientation	Side	Side	Side	Center	Center	Center	Side	Side	Side	Center	

**Table 4:** Energies of HOMO (E_HOMO_ in eV), LUMO (E_LUMO_ in eV), HOMO-LUMO gaps (E_H-L_ in eV), percent decrease in HOMO-LUMO gap (% ΔE_H-L_) and first hyperpolarizabilities (β_o_ in au) **endo-TM@X_12_Y_12_**.

Nanocage	Property	Transition Metals										
		Sc	Ti	V	Cr	Mn	Fe	Co	Ni	Cu	Zn	Bare
Al_12_N_12_	E_LUMO_	−2.67	−2.05	−2.68	−1.67	−1.78	−2.62	−2.13	−2.99	−2.08	−2.22	−2.54
	E_HOMO_	−4.34	−4.56	−4.23	−3.78	−4.06	−4.18	−4.04	−4.81	−4.24	−4.96	−6.47
	E_H-L_	1.67	2.51	1.56	2.12	2.28	1.55	1.91	1.82	2.16	2.74	3.93
	% ΔE_H-L_	57.6	36.0	60.4	46.2	42.0	60.5	51.4	53.7	45.0	30.4	0.0
	β_o_	17,590	480	279,495	681	1131	9631	11,691	100	2342	13	0
Al_12_P_12_	E_LUMO_	−3.42	−3.09	−3.26	−2.94	−3.06	−3.12	−3.40	−3.49	−3.06	−3.09	−3.36
	E_HOMO_	−4.95	−5.06	−5.20	−5.62	−6.06	−6.04	−5.12	−5.96	−5.64	−6.71	−6.75
	E_H-L_	1.53	1.97	1.94	2.68	3.00	2.92	1.72	2.47	2.59	3.62	3.39
	% ΔE_H-L_	54.8	41.9	42.8	20.8	11.4	13.7	49.4	27.3	23.7	−6.7	0.0
	β_o_	3954	2345	2773	403	3	72		302	702	0.82	0

**Table 5:** Polarizability (α in au) and first hyperpolarizability (β_o_ in au) of **endo-TM@X_12_Y_12_** complexes, calculated at LC-BLYP/6-31+G(d).

Transition Metal	Endo-TM@Al_12_N_12_		Endo-TM@Al_12_P_12_	
	α	β_o_	α	β_o_
Sc	486	17,590	664	3954
Ti	341	480	660	2345
V	522	279,495	624	2773
Cr	473	681	662	403
Mn	559	1131	663	3.0
Fe	327	9631	643	72
Co	348	11,691	648	2442
Ni	294	100	586	302
Cu	322	2342	618	702
Zn	326	13	626	0.82

**Table 6:** Comparison of nonlinear optical response of current work with already reported values over different metalide systems.

Complex	α_o_ (au)	β_o_ (au)	Ref.
endo-V@Al_12_N_12_	522	2.8 × 10^5^	This work
endo-Sc@Al_12_P_12_	664	3.9 × 10^3^	This work
Ti-C_19_	-	2.5 × 10^3^	[76]
Cu@r6-Al_12_N_12_	-	1.8 × 10^4^	[77]
Ni@Mg_12_O_12_	261	1.2 × 10^3^	[76]
Sc@r_4_-B_12_P_12_	612	4.4 × 10^4^	[78]

## Data Availability

All data are provided in the manuscript.
